# Lifespan Analysis of Dystrophic *mdx* Fast-Twitch Muscle Morphology and Its Impact on Contractile Function

**DOI:** 10.3389/fphys.2021.771499

**Published:** 2021-12-07

**Authors:** Leonit Kiriaev, Sindy Kueh, John W. Morley, Kathryn N. North, Peter J. Houweling, Stewart I. Head

**Affiliations:** ^1^Myogenica Laboratory, School of Medicine, Western Sydney University, Sydney, NSW, Australia; ^2^Muscle Research Group, Murdoch Children’s Research Institute, Melbourne, VIC, Australia

**Keywords:** Duchenne muscular dystrophy (DMD), fiber branching, extensor digitorum longus (EDL), eccentric contraction, *mdx*

## Abstract

Duchenne muscular dystrophy is caused by the absence of the protein dystrophin from skeletal muscle and is characterized by progressive cycles of necrosis/regeneration. Using the dystrophin deficient *mdx* mouse model, we studied the morphological and contractile chronology of dystrophic skeletal muscle pathology in fast-twitch Extensor Digitorum Longus muscles from animals 4–22 months of age containing 100% regenerated muscle fibers. Catastrophically, the older age groups lost ∼80% of their maximum force after one eccentric contraction (EC) of 20% strain with the greatest loss of ∼92% recorded in senescent 22-month-old *mdx* mice. In old age groups, there was minimal force recovery ∼24% after 120 min, correlated with a dramatic increase in the number and complexity of branched fibers. This data supports our two-phase model where a “tipping point” is reached when branched fibers rupture irrevocably on EC. These findings have important implications for pre-clinical drug studies and genetic rescue strategies.

## Introduction

In Duchenne Muscular dystrophy (DMD), the absence of dystrophin from skeletal muscle, triggers necrosis. The initial wave of necrosis is followed by regeneration of the skeletal muscle tissue and subsequent cycles of necrosis and regeneration (Moser, 1984; Wallace and McNally, 2009). The same pathology occurs in the most commonly used animal model of DMD, the dystrophin-deficient *mdx* mouse, where the long form of dystrophin is absent from the inner surface of the skeletal muscle sarcolemma (Bulfield et al., 1984; Coulton et al., 1988; Lefaucheur et al., 1995; Grounds et al., 2008; Ng et al., 2012). In the *mdx* mouse, many studies have shown that the predominantly fast-twitch Extensor Digitorum Longus (EDL) and Tibialis Anterior (TA) muscles are more susceptible to eccentric contraction (EC)-induced force deficit compared with dystrophin positive controls (Head et al., 1992; Moens et al., 1993; Dellorusso et al., 2001; Hogarth et al., 2017; Kiriaev et al., 2018). It is interesting to note that in the dystrophin deficient *mdx* mouse muscles, which lack type 2B (fast-twitch glycolytic) fibers (such as the soleus), are not susceptible to EC force deficits even though the absence of dystrophin in these muscles triggers waves of necrosis and regeneration such that the muscle fibers are completely replaced by 8 weeks of age (Duddy et al., 2015). The EC-induced drop in absolute force in fast-twitch muscles has been widely attributed to being the result of sarcolemmal damage and is commonly used as a model of membrane damage in dystrophinopathies (Head et al., 1992; Moens et al., 1993; Petrof et al., 1993; Williams et al., 1993; Faulkner et al., 1997; Brooks, 1998; Lynch et al., 2001a; Chan et al., 2007; Head, 2010, 2012; Chan and Head, 2011; Hakim et al., 2011; Han et al., 2011; Hakim and Duan, 2012; Kiriaev et al., 2018; Lindsay et al., 2018).

Studies designed to investigate possible treatments or cures for DMD commonly utilize the *mdx* mouse as a preclinical model to examine if the pharmaceutical or genetic intervention strategies prevent or reduce this EC force loss in dystrophin deficient fast-twitch skeletal muscle (Welch et al., 2007; Blaauw et al., 2008; Piers et al., 2011; Tinsley et al., 2011; Selsby et al., 2012; Aartsma-Rus and Muntoni, 2013; Fairclough et al., 2013; Rodino-Klapac et al., 2013; Roy et al., 2016). We believe these studies are flawed in cases where they assume that the EC force deficit is due to sarcolemmal rupture or tearing because there is a compelling body of evidence (Yeung et al., 2003, 2005; Whitehead et al., 2006b,^2008^, ^2010^; GervaìSio et al., 2008; Allen et al., 2016), demonstrating that in younger *mdx* mice, this EC deficit in the fast-twitch muscles is prevented by molecules that block stretch sensitive ion channels and also by exposing the dystrophic muscle to antioxidants before the EC (Pickering and Kiely, 2017). Current research suggests the presence of membrane impermeable dyes in *mdx* mice post EC is not conclusive evidence of membrane ruptures or rips. In skeletal muscle fibers from *mdx* mice, extracellular dye uptake is very small immediately after ECs and progressively increases up to at least 60 min post EC [see review by Allen et al. (2016)]. Both mechanosensitive channel blockers (Whitehead et al., 2006a) and the antioxidant N-acetyl cysteine (NAC; Whitehead et al., 2008) can prevent almost all the dye uptake, suggesting alternate pathways involving calcium entry through mechanosensitive channels and reactive oxygenated species (ROS) mediated membrane permeability. Recent support of this non-sarcolemmal damage pathway came from the work of Olthoff et al. (2018) where they demonstrated that EC force deficits could largely be reversed if the dystrophin deficient fast-twitch muscle was allowed to recover for a period of up to 120 min or if the period between repeated ECs was lengthened from the commonly used 3–5 min to 30 min. Additionally, Olthoff et al. (2018) showed that EC force deficit in younger 3-month-old *mdx* fast-twitch muscles was reduced by bath application of the antioxidant NAC or by genetically up-regulating an endogenous antioxidant. This adds more weight to Allen’s proposal [see Allen et al. (2016) for a review] that, in dystrophinopathies, muscles have a greater susceptibility to ROS-induced Ca^2+^ influx via abnormally functioning ion channels (Yeung et al., 2003, 2005) and it is the elevated [Ca^2+^]_in_ that triggers the cycles of necrosis/regeneration in dystrophinopathies.

Fast-twitch muscles from *mdx* mice lose up to 90% of their initial force generating capacity depending upon the EC protocol used (normally a series of between 3 and 120 contractions of varying length and velocity; Lindsay et al., 2020). During a series of ECs, the force deficit occurs incrementally with each EC rather than as an abrupt drop in force on the first EC which would be predicted if the sarcolemma was tearing or ripping. The exception to this is when you look at fast-twitch muscles from older (58–112 weeks) *mdx* mice where most of the force loss occurs abruptly during the first EC (Kiriaev et al., 2018). It is important to note that this is not an aging effect, as a similar phenomenon is not seen in the age matched littermate controls. We and others have shown that throughout the 30% shorter (Chamberlain et al., 2007; Li et al., 2009) lifespan of the *mdx* mouse, skeletal muscle undergoes continuous cycles of degeneration and regeneration (Coulton et al., 1988; Williams et al., 1993; Faulkner et al., 1997; Brooks, 1998; Lynch et al., 2001b; Chamberlain et al., 2007; Chan et al., 2007; Hakim et al., 2011; Han et al., 2011; Hakim and Duan, 2012; Lindsay et al., 2018; Massopust et al., 2020). Regenerated fibers are characterized by having central nuclei and as the number of cycles increases, so do the incidences of abnormal branched morphology (Head et al., 1990, 1992, 2004; Chan et al., 2007; Lovering et al., 2009; Friedrich et al., 2010; Pavlath, 2010; Pichavant and Pavlath, 2014; Kiriaev et al., 2018; Ben Larbi et al., 2021). As the *mdx* mouse ages, the branched fibers become more chaotic and bizarre in appearance, with a single continuous cytoplasmic syncytium being capable of supporting ten or more major branches (Head et al., 1992; Chan et al., 2007; Kiriaev et al., 2018). Once the number of branched fibers and their complexity of branching exceeds a threshold we refer to as “tipping point,” we and others have hypothesized that it is the branching, in and of itself, that weakens the muscle (Lovering et al., 2009; Head, 2010; Chan and Head, 2011; Buttgereit et al., 2013; Hernández-Ochoa et al., 2015; Pichavant et al., 2015; Iyer et al., 2017; Kiriaev et al., 2018; Massopust et al., 2020). Previously, our laboratory has shown this by looking at EDL muscles from the *mdx* dystrophic mouse from 2 weeks up to 112 weeks of age and it is only in the muscles greater than 6 months of age that there is a sudden loss of force with the first EC. Subsequent imaging of the muscle fibers *in–situ* and examination of enzymatically liberated single fibers show multiple examples of breaking and rupturing at branch points. In the present study, we use a strong EC protocol which produces force deficits in littermate control and *mdx* mice in adolescent to senescent aged groups and allow the muscles to recover for 120 min post-ECs to see if there is a non-recoverable force deficit correlated with fiber branching in dystrophic muscles.

## Materials and Methods

### Ethics Approval

Animal use was approved by the Western Sydney University Animal Care and Ethics Committee (A12907). These experiments were conducted in compliance with the animal ethics checklist and ethical principles under which the journal operates.

### Animals

The majority of previous dystrophic muscle function studies used separate colonies of wild-type control and dystrophic mice which have been inbred for over 25 years, introducing the possibility of new mutations to the groups.

In this study, littermates are bred to act as control animals for dystrophic mice. These are genetically more appropriate controls for dystrophin studies as both dystrophin negative and positive animals are from identical genetic backgrounds (Bellinger et al., 2009).

Male dystrophic mice with littermate controls were obtained from the Western Sydney University animal facility. The colony of dystrophic mice and littermate controls used in this study were second generation offspring crossed between female C57BL/10ScSn-Dmd*^mdx^* and male C57BL/10ScSn mice. Littermate controls were distinguished from dystrophic mice by genotyping.

Mice from five age groups were used in this study: 4, 9, 15, 18, and 22 months old where dystrophic muscles have undergone at least one round of necrosis/regeneration (Duddy et al., 2015). These age groups were selected to bridge the gap in dystrophic literature investigating muscle performance and recovery from contraction induced damage in adolescent to senescent mice (Flurkey et al., 2007). They were housed at a maximum of four to a cage in an environment-controlled room with a 12h-12h light-dark cycle. Standard rodent pellet chow (Gordon’s Specialty Stockfeeds, Yanderra, NSW, Australia) and water were available *ad libitum*. Basic enrichments such as nesting crinkle material and polyvinyl chloride pipe tube were provided.

A total of 71 male mice were used in this study (39 *mdx* mice and 32 littermate control mice). One hundred thirty-five EDL and 117 TA muscles collected for this study, not all of which were used in every procedure.

### Muscle Preparation

Mice were placed in an induction chamber and overdosed with isoflurane delivered at 4% in oxygen from a precision vaporizer. Animals were removed when they were not breathing, and a cervical dislocation was immediately carried out. Both the fast-twitch EDL and TA muscles were dissected from the hind limb which was submerged in oxygenated Krebs solution at all times during the dissection. TA muscles were dissected tendon to tendon, trimmed (excess tendons), and weighed. The dissected EDL muscle was tied by its tendons from one end to a dual force transducer/linear tissue puller (300 Muscle Lever; Aurora Scientific Instruments, Canada) and secured to a base at the other end using 6–0 silk sutures (Pearsalls Ltd, United Kingdom). Each muscle was then placed in a bath containing Krebs solution (also used as dissection solution) with a composition of (in mM): 4.75 KCl, 118 NaCl, 1.18 KH_2_PO_4_, 1.18 MgSO_4_, 24.8 NaHCO_3_, 2.5 CaCl_2_, 10 glucoses, 1 drop of antifoam, 0.1% fetal calf serum and bubbled continuously with carbogen (95% O_2_, 5% CO_2_) to maintain pH at 7.4. The muscle was stimulated by delivering a current between two parallel platinum electrodes using an electrical stimulator (701C stimulator; Aurora Scientific Instruments). All contractile procedures were designed, measured, and analyzed using the 615A Dynamic Muscle Control and Analysis software (Aurora Scientific Instruments). At the start of each experiment, the muscle was set to optimal length (*L*_o_) which produces maximal twitch force. Muscle dissection and experiments were conducted at room temperature (∼20–22^°^C).

### Initial Maximum Force and Contractile Protocols

An initial supramaximal stimulus was given at 125 Hz (1 ms pulses) for 1 s and force produced was recorded as *P*_o_ and the maximum force output of the muscle as *L*_o_. Pairs of EDL muscles from each animal were divided into two test groups that undergo either force frequency or isometric contraction protocols. Immediately after, all muscles were left to rest for 5 min, during which muscle length was reset to *L*_o_, followed by the EC and recovery procedures.

### Force Frequency Curve

Force frequency curves were generated for one set of muscles to measure contractile function. Trains of 1 ms pulse stimuli performed at different frequencies (2, 15, 25, 37.5, 50, 75, 100, 125, and 150 Hz) were given for 1 s. The forces produced were measured and a 30 s rest was allowed between each frequency. A sigmoid curve relating the muscle force (*P*) to the stimulation frequency (*f*) was fitted to these data.

The curve had the equation:


$$P=P_{\text{min}}+\frac{P_{\text{max}}-P_{\text{min}}}{1+{\left(\frac{K_{f}}{f}\right)}^{h}}$$

From the fitted parameters of the curve, the following contractile properties were obtained: force developed at minimal (*P*_min_) and maximal (*P*_max_) stimulation at the conclusion of the force frequency curve. Half-frequency (*K*_f_) is the frequency at which the force developed is halfway between (*P*_min_) and (*P*_max_) and Hill coefficient (*h*), which quantifies the slope of the muscle force frequency sigmoidal curve. These were used for population statistics.

### Isometric Contractions

The contralateral EDL muscle was subjected to 10 consecutive isometrics (fixed-length) supramaximal tetanic contractions, each lasting 1 s, separated by a 60 s rest, and then given a recovery contraction 5 min after the protocol (total of 11 contraction). Stimulus pulses within the 1 s tetanus were 1 ms in duration (width) and were delivered at a frequency of 125 Hz. This protocol is a modified version of the sequence used by Claflin and Brooks (2008). The force measured at each isometric contraction was expressed as a percentage of the force produced during the first (initial) contraction.

### Eccentric Contractions and Recovery

A series of eccentric (lengthening) contractions were then performed on each EDL where the contracted muscle was stretched 20% from *L*_*o*_. At *t* = 0 s, the muscle was stimulated via supramaximal pulses of 1 ms duration and 125 Hz frequency. At *t* = 0.9 s, after maximal isometric force was attained, each muscle was stretched 20% longer than their optimal length and held at this length for 2 s before returning to *L*_*o*_. Electrical stimulus was stopped at *t* = 5 s. The EC procedure was repeated six times with 3 min rest intervals. This is followed immediately by a recovery protocol. The force measured at each EC was expressed as a percentage of the force produced during the first (Initial) contraction. From the first EC data trace, baseline force was taken before and after the first EC to quantify baseline changes as a result of the first EC.

Upon completion of the six EC contraction sequence, recovery force was measured immediately (Post 0’) through isometric contractions given at 125 Hz (1 ms pulses) for 1 s and at the 20, 40, 60, 80, 100, and 120 min time points (Post 20’, Post 40’, etc.). These force values were then expressed as a percentage of the *P*_*o*_ measured before the six ECs. The recovery protocol is a modified version of the sequence used by Olthoff et al. (2018).

### Twitch Kinetics

Twitch kinetics were measured at three time points throughout the contractile protocol to compare differences between (1) pre-EC, (2) post EC, and (3) post-recovery kinetics. The twitches were performed at 2 Hz, for 1 ms (width), and 1 s duration. The first twitch taken from this response was used to measure kinetics immediately after initial maximum force (Pre) after the EC protocol (Post) and recovery protocol (Recov). The following parameters were collected: twitch force, half relaxation time (HRT), and time to peak (TTP).

### Muscle Stiffness

Stiffness is the resistance of an elastic body to deflection or deformation by an applied force. As an indicator of active muscle stiffness (during contraction), the change in force as the muscle is lengthened was measured during the first EC. Force was expressed as a percentage of the isometric force before stretching, and length was expressed as a percentage of optimum length. To estimate muscle stiffness, the change in muscle force was divided by the change in muscle length (as a percentage of *L*_*o*_) during the first EC.

### Work Done

Work is an energy quantity given by force multiplied by distance. This measurement was used to provide a quantitative estimate of the eccentric damage-inducing forces. Work done to stretch the muscle was calculated from the force tracings through multiplying the area underneath the lengthening phase of the force tracing with the velocity of lengthening.

### Muscle Mass and Cross-Sectional Area

After contractile procedures were completed, the EDL muscle was removed from the organ bath and tendons trimmed. Both the EDL and TA muscles were blotted lightly (Whatmans filter paper DE81 grade) and weighed using an analytical balance (GR Series analytical electronic balance).

Physiological cross-sectional area (PCSA) was calculated by dividing the muscle mass by the product of its length and mammalian muscle density. Specific muscle force was obtained through dividing raw force values by cross-sectional area (CSA). When normalizing force using a calculation of PCSA in mouse EDL, many studies use a correction factor to allow for fiber length (*L*_*f*_). We chose not to in the present study because:

(i) There is considerable variation in the literature as to the value of *L*_*f*_/*L*_*o*_ in mouse EDL muscle with a ratio of 0.44, 0.68, 0.75, and 0.85 being used (Crow and Kushmerick, 1982; Brooks and Faulkner, 1988; James et al., 1995; Tallis et al., 2012). Depending on which one is adopted, widely varying values for specific force will be obtained. In this study, we were not primarily concerned with the actual values of the specific force; we were mainly interested in seeing whether it differed between the ages and genotype.

(ii) As we show here, branched fibers are found in the dystrophic muscles, and the measurement of fiber length becomes especially problematic in branched fibers. The application of a uniform *L_*f*_/L_*o*_* across the whole muscle might not be valid due to the different geometry of branched fibers and unbranched fibers. It is not clear how the length of the fiber would be defined. It is possible that some other method of estimating total fiber CSA is necessary in a muscle containing branched fibers.

However, we did normalize forces with respect to an estimate of PCSA according to the equation, CSA = MM/(*L*_*o*_ × *D*), where MM is the muscle mass, *L*_*o*_ is the optimal length, and *D* is the density of skeletal muscle (1.06 g/cm^3^) to enable us to compare muscle of differing sizes and weights (Hayes and Williams, 1998). In healthy rodent hind limb muscles, maximal tension was found to be directly proportional to calculated PCSA (Powell et al., 1984). However, this method was still not ideal as we were assuming that the muscle density is unaltered by fat and connective tissue infiltration, which is accumulated in the old dystrophic animals (Pastoret and Sebille, 1995).

### Skeletal Muscle Single Fiber Enzymatic Isolation and Morphology

Following contractile procedures and weighing, EDL muscles were digested in Krebs solution (without FCS) containing 3 mg/ml collagenase type IV A (Sigma Aldrich, United States), gently bubbled with carbogen (95% O_2_, 5% CO_2_), and maintained at 37^°^C. After 25 min, the muscle was removed from solution, rinsed in Krebs solution containing 0.1% fetal calf serum, and placed in a relaxing solution with the following composition (mM): 117 K^+^, 36 Na^+^, 1 Mg^2+^, 60 Hepes, 8 ATP, and 50 EGTA (Note: internal solution due to chemically skinning by high EGTA concentration). Each muscle was then gently agitated using pipette suction, releasing individual fibers from the muscle mass. Using a pipette, 0.5 ml of solution was drawn, placed on a glass slide for examination, and photographs of dissociated fibers taken. During counting, each fiber and its associated branches are counted as one fiber. In our earlier paper (Head et al., 1990), we demonstrated (using intracellular dye injection) that the branches are part of the same sarcoplasmic compartment. Moreover, if a micropipette is used to stimulate any portion of the branched syncytium, the whole branched fiber complex contracts. In instances where a long fiber covered several fields of the microscope view, a series of overlapping photomicrographs were taken, and these were stitched together using the Coral draw graphic package.

A total of 11,657 fibers from 65 EDL muscles were counted: 7,119 fibers from 34 controls and 4,538 from 31 dystrophic muscles. Only intact fibers with no evidence of digestion damage were selected for counting.

### Statistical Analyses

Data was presented as means ± SD. Differences occurring between genotypes and age groups were assessed by two-way ANOVA with genotype being one fixed effect and age groups the other. *Post hoc* analysis was performed using Sidak’s multiple comparisons test. All tests were conducted at a significance level of 5%. All statistical tests and curve fitting were performed using a statistical software package Prism Version 7 (GraphPad, CA, United States).

## Results

### Degree of Fiber Branching and Complexity With Age

**FIGURE 1:**
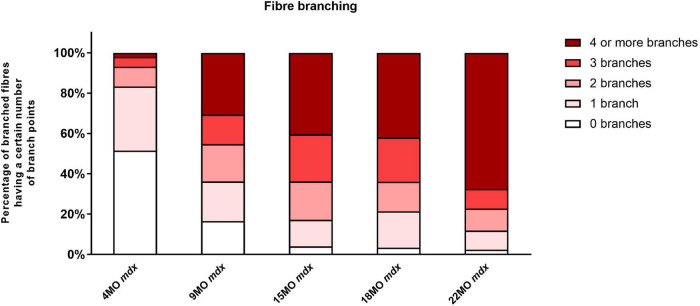
The degree of fiber branching in extensor digitorum longus (EDL) muscles as a percentage of all fibers by categorizing them based on number of branch points. Fibers from littermate controls were omitted due to very low presence of fiber branching, particularly, less than 1%. Note this is a bar graph of all fibers counted and as such is an absolute measure, all photomicrographs in Figures 9–11 were taken from these fiber counts. A total of 11,657 fibers from 65 EDL muscles were counted: 7,119 fibers from 34 controls, and 4,538 fibers from 31 *mdx* muscles. For the 4-month group: *n* = 1,514 control *n* = 1,030 *mdx* fibers; 9-month group: *n* = 1,320 control *n* = 1,130 *mdx* fibers; 15-month group: *n* = 1,615 control *n* = 1,263 *mdx* fibers; 18-month group: *n* = 1,435 control *n* = 605 *mdx* fibers; and 22 month group: *n* = 1,235 control *n* = 511 *mdx* fibers.

The number of branched fibers and complexity of fiber branching within a single branched fiber syncytium found in *mdx* EDL muscles is shown in Figure 1 (note these counts represent all fibers counted and there are no error bars). The evidence (Bell and Conen, 1968; Schmalbruch, 1984; Head et al., 1990, 1992; Chan et al., 2007; Lovering et al., 2009; Friedrich et al., 2010; Pavlath, 2010; Chan and Head, 2011; Buttgereit et al., 2013; Faber et al., 2014; Pichavant and Pavlath, 2014; Duddy et al., 2015; Kiriaev et al., 2018), now overwhelming, supports the findings shown in Figure 1 that as the *mdx* animals age, the number and complexity of branched fibers increases dramatically. Single enzymatically isolated EDL fibers from littermate control animals showed between <1% and ∼3% branching in all age groups (consisted with reports from other studies; Schmalbruch, 1984; Head et al., 1990, 1992, 2014; Bockhold et al., 1998; Chan et al., 2007; Head, 2010; Pichavant and Pavlath, 2014; Kiriaev et al., 2018). In the 4-month *mdx*, 48% of all fibers counted were branched with the branching relatively simple with 41% of branched fibers having one or two branches per fiber. By the time they reach 9 months of age, 83% of fibers contain branches. Of these, 38% of fibers contain one or two branches and 45% of fibers were with three or more branches per fiber syncytium. At 15 and 18 months of age, dystrophic EDL muscles have 96% branched fibers, of which 64% have three or more branches per fiber syncytium and 32% having one or two branches. At 22 months of age, the majority of *mdx* EDL fibers contained branches with 77% showing three or more branches per fiber.

### Mass, Length, and Cross-Sectional Area

**FIGURE 2:**
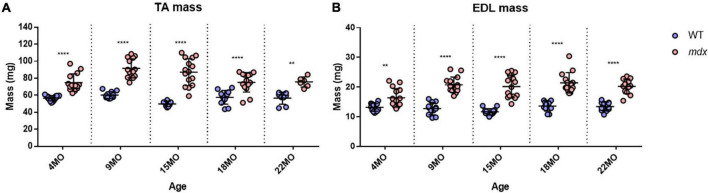
Muscle mass for all age groups. **(A)** Interleaved scatterplot of tibialis anterior (TA) muscle mass for 4-month (*n* = 14 control; *n* = 14 *mdx*), 9-month (*n* = 12 control; *n* = 14 *mdx*), 15-month (*n* = 8 control; *n* = 15 *mdx*), 18-month (*n* = 12 control; *n* = 14 *mdx*), and 22-month (*n* = 8 control; *n* = 6 *mdx*) age groups. **(B)** Interleaved scatterplots of EDL muscle mass for 4-month (*n* = 14 control; *n* = 15 *mdx*), 9-month (*n* = 12 control; *n* = 15 *mdx*), 15-month (*n* = 14 control; *n* = 15 *mdx*), 18-month (*n* = 12 control; *n* = 14 *mdx*), and 22-month (*n* = 12 control; *n* = 12 *mdx*) age groups. In each group the horizontal line indicates the mean value ± SD. There were no statistical differences between age groups, statistical differences displayed within graphs are differences between genotypes assessed by two-way ANOVA, *post hoc* analysis using Sidak’s multiple comparisons test. ****P < 0.0001 and **0.001 < *P* < 0.01.

Tibialis Anterior muscles were heavier for *mdx* animals compared to littermate controls at all age groups (Figure 2A) with peak differences in muscle mass occurring at 15 months [MD 37.3, 95% CI (26.39, 48.21), *P* < 0.0001] compared to 4 months [MD 18.34, 95% CI (8.92, 27.76), *P* < 0.0001] and 22 months [MD 19.12, 95% CI (5.65, 32.58), *P* = 0.0016] age groups. These findings and a lack of age effects on muscle mass are consistent with dystrophic TA studies published previously (Sacco et al., 1992; Quinlan et al., 1992; Pastoret and Sebille, 1995; Dellorusso et al., 2001; Hakim et al., 2011). The same increase in dystrophic muscle mass can also be seen in the EDL (Figure 2B) with peak differences likewise occurring at 15 months of age [MD 8.52, 95% CI (6.09, 10.96), *P* < 0.0001] compared to 4 month [MD 3.18, 95% CI (0.74, 5.62), *P* = 0.0045] and 22 month [MD 6.75, 95% CI (4.08, 9.43), *P* < 0.0001] age groups. Muscle hypertrophy in *mdx* muscle is a recognized feature (Deconinck et al., 1997; Faulkner et al., 1997; Hayes and Williams, 1998; Lynch et al., 2001b; Hakim et al., 2011; Han et al., 2011; Hakim and Duan, 2012; Capogrosso et al., 2017; Kiriaev et al., 2018; Lindsay et al., 2018) and can largely be attributed to the fiber branching which occurs in the regenerated dystrophic fibers (Head et al., 1992; Faber et al., 2014; Pichavant and Pavlath, 2014; Kiriaev et al., 2018). It should be noted that both TA and EDL control muscle mass has remained consistent with age and there was no evidence of sarcopenia up to 22 months of age. Other properties for the EDL, such as length and PCSA for all age groups, are shown in Table 1. EDL muscle length remained the same regardless of genotype or age, hence when PCSA was calculated for each muscle, the differences can be attributed to those seen in EDL muscle mass.

**TABLE 1:** Statistical analyses and sample size of muscle properties, force frequency parameters, and kinetics for EDL muscles across all age groups.

		4MO			9MO			15MO			18MO			22MO		
		Control	*mdx*	*P*-value	Control	*mdx*	*P*-value	Control	*mdx*	*P*-value	Control	*mdx*	*P*-value	Control	*mdx*	*P*-value
A	Muscle length (mm)	13.86 ± 0.23	13.86 ± 0.23	NS	13.88 ± 0.31	13.88 ± 0.22	NS	14 ± 0.28	14 ± 0.27	NS	13.85 ± 0.58	13.84 ± 0.54	NS	14.08 ± 0.20	14.21 ± 0.26	NS
	PCSA (mm^2^)	0.90 ± 0.09	1.11 ± 0.22	**	0.87 ± 0.15	1.41 ± 0.17	****	0.79 ± 0.07	1.36 ± 0.25	****	0.93 ± 0.14	1.45 ± 0.28	****	0.90 ± 0.10	1.35 ± 0.16	****
	*N* (muscles)	14	15	–	12	15	–	14	15	–	12	14	–	12	12	–
B	Half frequency (Hz)	32.05 ± 3.20	30.67 ± 3.03	NS	35.13 ± 3.56	34.62 ± 2.58	NS	35.49 ± 1.97	34.00 ± 2.74	NS	31.75 ± 2.69	33.80 ± 2.62	NS	31.72 ± 3.52	34.10 ± 1.66	NS
	Hill coefficient	4.28 ± 0.32	4.15 ± 0.34	NS	4.66 ± 0.34	4.64 ± 0.38	NS	4.50 ± 0.26	4.49 ± 0.27	NS	4.60 ± 0.34	4.89 ± 0.52	NS	4.73 ± 0.70	4.49 ± 0.46	NS
	*N* (muscles)	7	7	–	6	7	–	7	6	–	6	7	–	6	7	–
C	Pre EC HRT (ms)	19.5 ± 2.35	23.61 ± 3.07	***	17.68 ± 1.51	18.72 ± 3.62	NS	17.98 ± 2.03	17.71 ± 1.34	NS	18.71 ± 1.25	17.77 ± 1.93	NS	20.50 ± 2.94	19.00 ± 2.07	NS
	Post EC HRT (ms)	17.07 ± 1.28	26.44 ± 4.15	****	17.86 ± 1.78	26.54 ± 6.38	****	18.15 ± 1.36	26.54 ± 6.82	****	18.00 ± 1.09	22.13 ± 4.40	**	18.50 ± 1.39	21.67 ± 2.57	NS
	Recov EC HRT (ms)	16.71 ± 1.28	24.00 ± 3.71	****	15.96 ± 1.25	20.67 ± 2.43	****	16.36 ± 1.49	20.50 ± 2.16	****	16.88 ± 1.43	22.00 ± 2.15	****	16.40 ± 0.81	21.64 ± 1.72	****
	Pre EC TTP (ms)	27.96 ± 2.14	29.17 ± 1.77	NS	28.00 ± 1.40	25.94 ± 1.62	*	29.50 ± 1.23	26.46 ± 1.91	****	29.08 ± 1.76	24.96 ± 1.80	****	29.50 ± 2.30	26.21 ± 1.27	***
	Post EC TTP (ms)	23.68 ± 1.48	26.72 ± 1.35	***	24.27 ± 1.42	25.42 ± 1.91	NS	26.10 ± 1.54	24.43 ± 2.53	NS	24.29 ± 1.39	23.5 ± 2.65	NS	24.50 ± 1.99	24.21 ± 1.05	NS
	Recov EC TTP (ms)	22.25 ± 1.68	25.28 ± 1.48	****	22.00 ± 1.41	23.71 ± 1.01	*	24.50 ± 1.08	23.50 ± 1.72	NS	22.50 ± 1.38	22.96 ± 1.45	NS	21.90 ± 1.31	23.64 ± 0.81	*
	Pre EC HRT/TTP	0.70 ± 0.05	0.81 ± 0.09	**	0.63 ± 0.04	0.72 ± 0.11	*	0.61 ± 0.07	0.67 ± 0.04	NS	0.64 ± 0.04	0.71 ± 0.08	NS	0.70 ± 0.10	0.72 ± 0.06	NS
	Post EC HRT/TTP	0.72 ± 0.03	0.99 ± 0.13	****	0.74 ± 0.04	1.04 ± 0.21	****	0.70 ± 0.05	1.08 ± 0.21	****	0.74 ± 0.02	0.94 ± 0.16	***	0.76 ± 0.07	0.90 ± 0.12	**
	Recov EC HRT/TTP	0.75 ± 0.03	0.95 ± 0.11	****	0.73 ± 0.03	0.87 ± 0.07	****	0.67 ± 0.06	0.87 ± 0.09	****	0.75 ± 0.02	0.96 ± 0.10	****	0.75 ± 0.05	0.92 ± 0.06	****
	*N* (muscles)	14	9	–	11	16	–	14	14	–	12	13	–	10	12	–

Muscle properties (Row section A): Muscle length and calculated physiological cross-sectional area. Force frequency parameters (Row section B): Half frequency and Hill coefficient. Kinetics (Row section C): HRT, Half relaxation time; TTP, Time to peak; and HRT/TTP ratio. Twitch kinetics were measured (1) pre-EC, (2) post-EC, and (3) after recovery from the EC protocol. All data shown are mean ± SD. No statistical differences were found between age groups, P-values show genotype effects assessed by two-way ANOVA, post hoc analysis using Sidak’s multiple comparisons test. ****P < 0.0001, ***0.0001 < P < 0.001, **0.001 < P < 0.01, *0.01 < P < 0.05, and NS labeled for no statistical significance.

### Maximal Tetanic and Twitch Force

**FIGURE 3:**
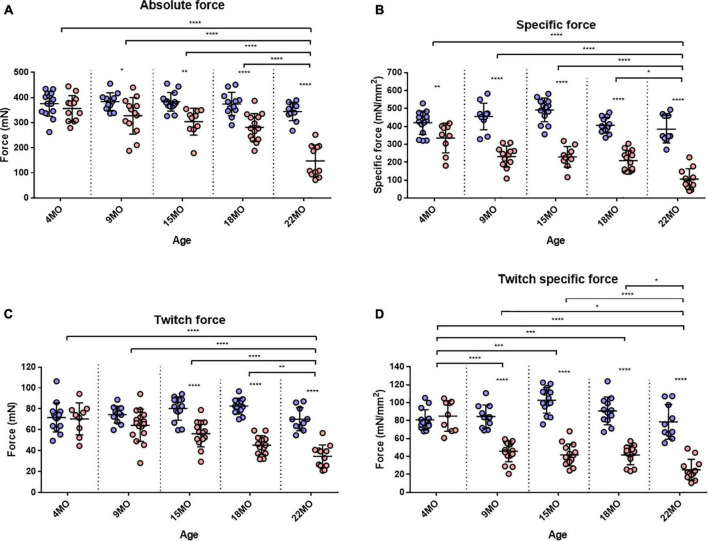
Maximum tetanic and twitch force. **(A)** Interleaved scatterplot of the maximum absolute force generated by EDL muscles across 4-month (*n* = 14 control; *n* = 12 *mdx*), 9-month (*n* = 11 control; *n* = 13 *mdx*), 15-month (*n* = 14 control; *n* = 10 *mdx*), 18-month (*n* = 12 control; *n* = 14 *mdx*), and 22-month (*n* = 10 control; *n* = 11 *mdx*) age groups. **(B)** Interleaved scatterplot of the maximum specific force (force per physiological cross-sectional area) generated by EDL muscles across 4-month (*n* = 14 control; *n* = 9 *mdx*), 9-month (*n* = 11 control; *n* = 12 *mdx*), 15-month (*n* = 14 control; *n* = 10 *mdx*), 18-month (*n* = 12 control; *n* = 14 *mdx*), and 22-month (*n* = 10 control; *n* = 11 *mdx*) age groups. **(C)** Interleaved scatterplot of the twitch force generated by EDL muscles across 4-month (*n* = 14 control; *n* = 9 *mdx*), 9-month (*n* = 11 control; *n* = 16 *mdx*), 15-month (*n* = 14 control; *n* = 14 *mdx*), 18-month (*n* = 12 control; *n* = 13 *mdx*), and 22-month (*n* = 10 control; *n* = 12 *mdx*) age groups. **(D)** Interleaved scatterplot of the twitch specific force generated by EDL muscles across 4-month (*n* = 14 control; *n* = 9 *mdx*), 9-month (*n* = 11 control; *n* = 16 *mdx*), 15-month (*n* = 14 control; *n* = 14 *mdx*), 18-month (*n* = 12 control; *n* = 13 *mdx*), and 22-month (*n* = 10 control; *n* = 12 *mdx*) age groups. In each group the horizontal line indicates the mean value ± SD. Statistical differences between age groups are displayed above the graph, statistical differences displayed within graphs are differences between genotypes assessed by two-way ANOVA, *post hoc* analysis using Sidak’s multiple comparisons test. ****P < 0.0001, ***0.0001 < *P* < 0.001, **0.001 < *P* < 0.01, and *0.01 < *P* < 0.05.

The EDL muscle isometric maximum force production (*P*_0_) for all age groups is presented in Figure 3A. 4-month-old mice showed no significant differences in force production between dystrophic and littermate control muscles. However, as the animals age from 9 to 22 months, the maximum force generated by the dystrophic EDL is significantly less than age matched littermate controls. The low force output is most pronounced in the oldest mice, where EDL muscles from 18 to 22-month-old *mdx* mice produced ∼25 and ∼57% less force, respectively, than controls [MD −92.95, 95% CI (−146.2, −39.73), *P* < 0.0001] [MD −197.1, 95% CI (−256.2, −138), *P* < 0.0001]. Interestingly, the 22-month-old dystrophic cohort produced significantly less force compared to all other age groups [*F* (4, 111) = 9.26, *P* < 0.0001]. When each EDL muscle’s force was corrected for PCSA (Figure 3B), the 22-month-old dystrophic cohort still produced significantly less specific force compared to dystrophic muscles from all other age groups [*F* (4, 107) = 7.85, *P* < 0.0001]. In all the age groups looked at, *mdx* EDL muscles produced significantly less specific force compared to littermate control animals. At 4 months of age, the *mdx* muscles produce ∼20% less force compared to controls [MD −84.92, 95% CI (−156.6, −13.28), *P* = 0.012]. This deficit increases at 15 months with *mdx* muscles producing ∼54% less force compared to controls [MD −262.5, 95% CI (−331.9, −193), *P* < 0.0001] and peaks at 22 months of age where dystrophic EDL muscles generated ∼73% less force than controls [MD −279.4, 95% CI (−352.7, −206.1), *P* < 0.0001]. This decline in specific force correlates with the increase in number and complexity of branched EDL fibers in the dystrophic mice (Figure 1).

Twitch forces for all groups are shown in Figure 3C. For 4 and 9-month-old mice, there was no significant difference in twitch force between genotypes. However, with increasing age, dystrophic muscles produce significantly less force than age matched littermate control muscle. Differences in EDL twitch force between aged *mdx* mice and littermate controls were as follows: 15 months ∼30% [MD −24.04, 95% CI (−35.8, −12.28), *P* < 0.0001], 18 months ∼45% [MD −37.38, 95% CI (−49.83, −24.92), *P* < 0.0001] and 22 months ∼51% [MD −35.51, 95% CI (−48.84, −22.19), *P* < 0.0001]. The twitch force in 22-month-old dystrophic mice was significantly less when compared to other age groups [*F* (4, 115) = 4.99, *P* < 0.0001]. Figure 3D shows twitch force values corrected for CSA. While the 4-month dystrophic EDL produced the same specific twitch force as littermate controls, from 9 to 22 months, the dystrophic EDL muscles produced significantly less specific twitch force. [*F* (4, 115) = 20.8, *P* < 0.0001]. This decrease in specific twitch force correlates with the increase in number and complexity of branched fibers (Figure 1).

### Force Frequency Parameters Are Not Significantly Different With Respect to Age and Genotype

**FIGURE 4:**
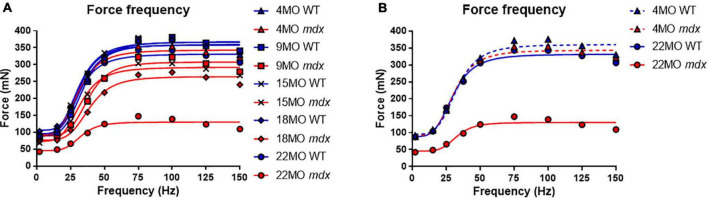
Force frequency curves. **(A)** Aggregated force frequency curves from EDL muscles to visualize differences between genotype across 4-month (*n* = 7 control; *n* = 7 *mdx*), 9-month (*n* = 6 control; *n* = 7 *mdx*), 15-month (*n* = 7 control; *n* = 6 *mdx*), 18-month (*n* = 6 control; *n* = 7 *mdx*), and 22-month (*n* = 6 control; *n* = 7 *mdx*) age groups. Muscle absolute force was measured at different stimulation frequencies and a sigmoidal curve (lines) was fitted to the data points (see section “Materials and Methods”), contractile properties were generated from these curves (see Table 1). **(B)** For clarity 4-month and 22-month old age groups have been isolated to highlight the shape and slope of these force frequency curves. Data shown in these curves are mean only, SD was omitted for visual clarity.

Force frequency curves were generated for all age groups and genotypes (Figure 4A). For clarity, in Figure 4B, only curves for 4 and 22-months-old EDL muscles are shown. As our group have reported previously for the *mdx* EDL (Williams et al., 1993; Chan et al., 2007; Kiriaev et al., 2018), there was no significant difference in half frequency or Hill coefficient with respect to genotype or age (Table 1). Figure 4 visualizes the decline in force with age in dystrophic *mdx* EDL. As we note in the discussion, this age-related decline in force output from the dystrophic EDL muscles is strongly correlated with the development of branched fibers (Figure 1).

### Isometric Force Loss in Older *mdx* Extensor Digitorum Longus Muscles Is Correlated With Increased Fiber Branching

**FIGURE 5:**
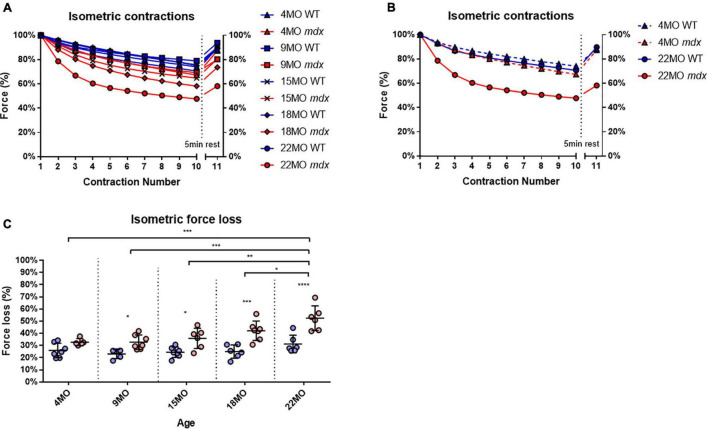
Percentage force changes throughout repeat isometric contractions and recovery. **(A)** Shows the change in isometric force expressed as a percentage of starting force across 10 consecutive contractions and an 11th recovery contraction after 5 min for all age groups. **(B)** For clarity, 4-month and 22-month old age groups have been isolated to highlight the differences in isometric force loss and recovery across these contractions. Data shown in these curves are mean only; SD was omitted for visual clarity. **(C)** An interleaved scatterplot of the cumulative force loss by the EDL across 4-month (*n* = 7 control; *n* = 5 *mdx*), 9-month (*n* = 5 control; *n* = 7 *mdx*), 15-month (*n* = 7 control; *n* = 6 *mdx*), 18-month (*n* = 6 control; *n* = 7 *mdx*), and 22-month (*n* = 6 control; *n* = 6 *mdx*) age groups (Note: these numbers apply to **A–C**). In each interleaved scatterplot group the horizontal line indicates the mean value ± SD. Statistical differences between age groups are displayed above the graph, statistical differences displayed within graphs are differences between genotypes assessed by two-way ANOVA, *post hoc* analysis using Sidak’s multiple comparisons test. ****P < 0.0001, ***0.0001 < *P* < 0.001, **0.001 < *P* < 0.01, and *0.01 < *P* < 0.05.

Extensor digitorum longus muscles from each age group and genotype were subjected to a series of 10 maximal isometric contractions at 1-min intervals, and the final “recovery” contraction (no 0.11) was given after a 5-min rest interval. To enable change in force comparisons to be made between EDL muscles from different age groups and genotype, the isometric force was normalized to the first isometric contraction. All the age group and genotype data are shown in Figure 5A, while for clarity, Figure 5B shows only data from the 4 and 22-month-age groups. With increasing age in dystrophic EDL muscles, there is a concomitant increase in isometric force loss over the 10 contractions and reduced force recovery on the 11th contraction (Figure 5). Figure 5B shows that the isometric response in EDL muscles from 4-month-old dystrophic mice is similar to age matched littermate controls. In contrast, by 22 months of age, *mdx* EDL muscles lost ∼52% of their starting force, and only recovered to ∼60% compared to a loss of ∼31% in 22-month-old control animals, which eventually recovered close to ∼100% [MD 21.28, 95% CI (11.09, 31.48), *P* < 0.0001]. It is important to note that dystrophin is absent in both 4 and 22-month-old *mdx* EDL muscles, and the age-matched littermate controls discount an aging effect, leading us to propose that once there are a significant number of complexed branched fibers present in the *mdx* EDL, there is a causative connection between the isometric force loss and the degree of fiber branching Figure 1 correlated with Figure 5.

### Eccentric Force Deficits

**FIGURE 6:**
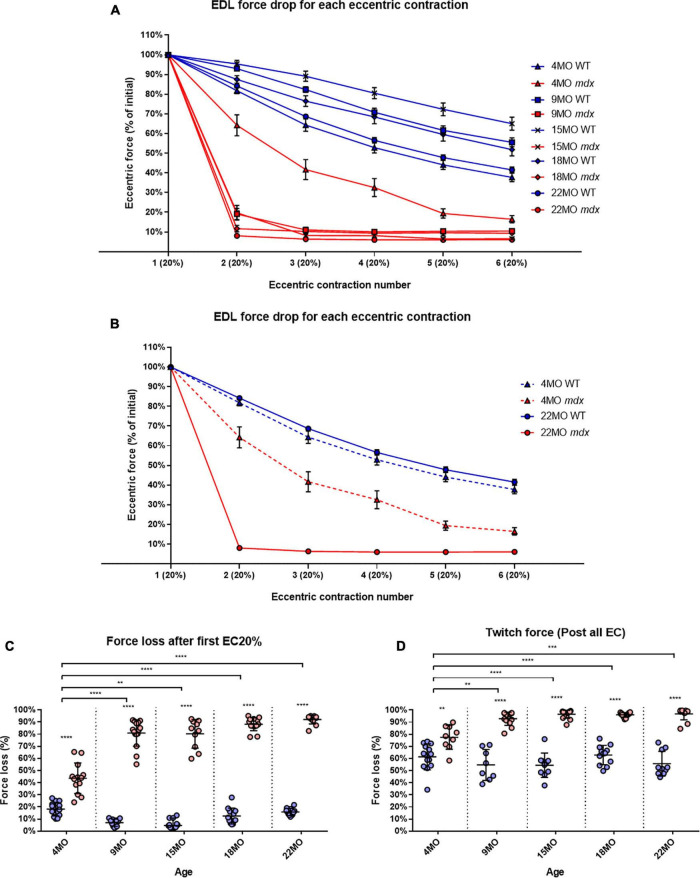
Percentage force loss resulting from a series of six eccentric contractions (ECs) at 20% excursion from *L*_*o*_. Forces were normalized in each group with *P*_max_ = 100%. **(A)** XY plot of EDL force loss for *mdx* mice and littermate controls across all age groups. **(B)** For clarity 4 month and 22-month-old age groups have been isolated to highlight the differences in eccentric force loss across these contractions. Data shown in these curves are mean ± SD. **(C)** Interleaved scatterplots of the force loss after the first 20% EC across 4-month (*n* = 14 control; *n* = 13 *mdx*), 9-month (*n* = 11 control; *n* = 15 *mdx*), 15-month (*n* = 12 control; *n* = 10 *mdx*), 18-month (*n* = 12 control; *n* = 13 *mdx*), and 22-month (*n* = 12 control; *n* = 12 *mdx*) age groups (Note: these numbers apply to **A–C**). **(D)** Interleaved scatterplots of the twitch force loss after all six EC across 4-month (*n* = 14 control; *n* = 9 *mdx*), 9-month (*n* = 11 control; *n* = 16 *mdx*), 15-month (*n* = 14 control; *n* = 14 *mdx*), 18-month (*n* = 12 control; *n* = 13 *mdx*), and 22-month (*n* = 10 control; *n* = 12 *mdx*) age groups. For interleaved scatterplots in **(C,D)**, the horizontal line indicates the mean value ± SD. Statistical differences between age groups are displayed above the graph, statistical differences displayed within graphs are differences between genotypes assessed by two-way ANOVA, *post hoc* analysis using Sidak’s multiple comparisons test. ****P < 0.0001, ***0.0001 < *P* < 0.001, and **0.001 < *P* < 0.01.

During a series of six ECs, there was a uniform graded force deficit in dystrophic EDL muscles from 4 month old *mdx*, however, in *mdx* mice from 9 to 22 months, the majority of the EC force deficit in EDL occurred on the first contraction. The isometric force drops after each EC is normalized to starting force for all age groups (Figure 6). Figure 6B shows data from the 4 and 22-month-old age groups. In all age groups, *mdx* EDL muscles lost more force during the EC protocol than age-matched littermate controls (Figure 6A). However, the *mdx* EDL muscles in the 4-month group were the only dystrophic muscles to lose force in graded steps during the six ECs, resembling the step-like force deficits produced in age matched littermate control EDL throughout the EC protocol (Figures 6A,B). The *mdx* 4-month EDL lost ∼44% of starting force compared to ∼18% in controls on the first EC [MD 25.4, 95% CI (17.62, 33.18), *P* < 0.0001]. In contrast to the 4-month *mdx* EDL, dystrophic EDL muscles from the older 9 to 22-month age groups had ≥80% force deficit after the first EC in the series, with the greatest lost at ∼92% for 22-month-old *mdx* mice compared with ∼16% in age matched littermate controls [MD 76.29, 95% CI (68.05, 84.54), *P* < 0.0001] (Figure 6C). In the older *mdx*, the 9 to 22-month-old age groups, the final five EC were basically passive stretches due to the negligible force output. Again, the correlation between the catastrophic force loss experienced by the 9 to 22-month *mdx* cohort and increases in fiber branching is striking.

In line with the tetanic force data, EDL twitch force from the older 9 to 22-month *mdx* mice dropped dramatically after the EC protocol (Figure 6D). EDL muscles from 4-month *mdx* animals lost an average of ∼78% of their starting twitch force compared with ∼61% in controls after the EC protocol [MD 16.19, 95% CI (6.92, 25.45), *P* = 0.0016], 9-month *mdx* animals lost ∼93% of their starting twitch force compared to ∼55% in controls after the EC protocol [MD 38.16, 95% CI (28.48, 47.83), *P* < 0.0001]. In the aged *mdx* cohorts, EDL muscle twitches barely produced any force reflecting the consequence to force loss due to EC-induced damage in Figures 6A-C. Again, the largest force deficit occurred in 22-month *mdx* mice losing ∼97% of the pre-EC twitch force compared with ∼56% in littermate controls [MD 41.14, 95% CI (32.06, 50.21), *P* < 0.0001].

### Rapid Recovery From Eccentric Force Loss Is Seen in 4-Month *mdx* but to a Lesser Degree as the *mdx* Mice Ages

**FIGURE 7:**
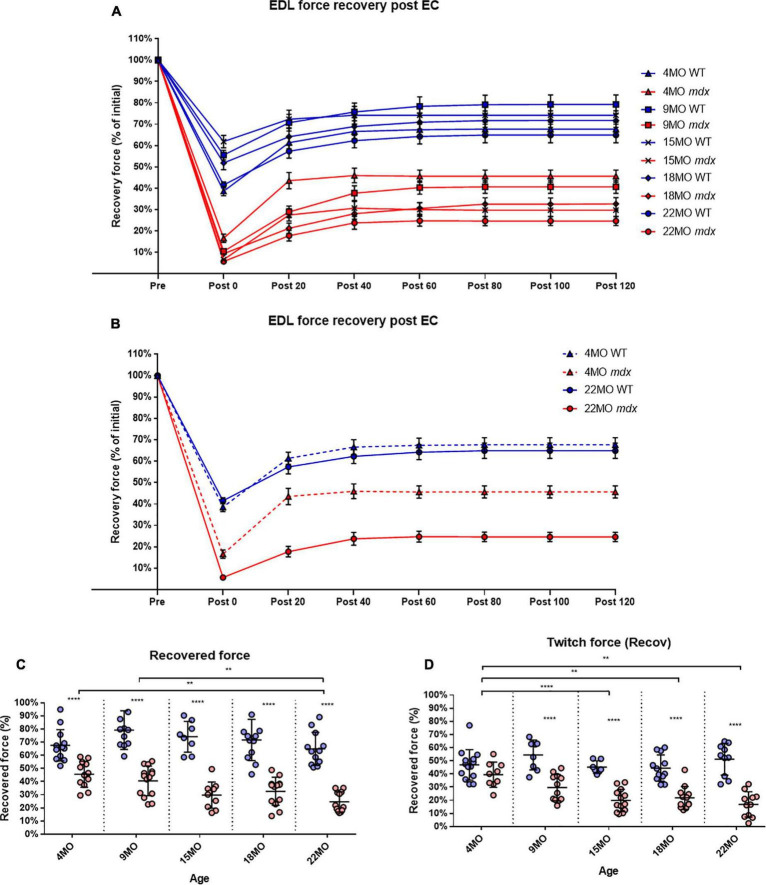
Percentage force recovery for up to 120 min post EC protocol. Forces were normalized in each group with *P*_max_ = 100%. **(A)** XY plot of post EC EDL force recovery over 120 min at 20-min intervals for *mdx* and littermate controls across all age groups. **(B)** For clarity, 4 and 22-month-old age groups have been isolated to highlight the differences in post EC recovery across these contractions. Data shown in these curves are mean ± SD. **(C)** Interleaved scatterplots of the recovery at 120 min across 4-month (*n* = 13 control; *n* = 12 *mdx*), 9-month (*n* = 11 control; *n* = 14 *mdx*), 15-month (*n* = 8 control; *n* = 10 *mdx*), 18-month (*n* = 12 control; *n* = 13 *mdx*), and 22-month (*n* = 12 control; *n* = 12 *mdx*) age groups (Note: these numbers apply to **A–C**). **(D)** Interleaved scatterplots of the twitch force recovery at 120 min across 4-month (*n* = 14 control; *n* = 9 *mdx*), 9-month (*n* = 11 control; *n* = 12 *mdx*), 15-month (*n* = 7 control; *n* = 13 *mdx*), 18-month (*n* = 12 control; *n* = 12 *mdx*), and 22-month (*n* = 10 control; *n* = 11 *mdx*) age groups. For interleaved scatterplots in **(C,D)**, the horizontal line indicates the mean value ± SD. Statistical differences between age groups are displayed above the graph, statistical differences displayed within graphs are differences between genotypes assessed by two-way ANOVA, *post hoc* analysis using Sidak’s multiple comparisons test. ****P < 0.0001, **0.001 < *P* < 0.01.

To measure the amount of recovery attributable to non-sarcolemmal damage, we used the rationale of Olthoff et al. (2018), measuring the maximum force every 20 min for 2 h post-EC protocol (Figure 7A). Our EC was strong enough to cause fiber damage in control mice where there was a 20−35% non-recoverable force loss. Figure 7B illustrates the EDL recovery post-EC at 20-min intervals for 4 and 22-month-old mice. EDL from 4-month-old mdx mice showed the greatest amount of recovery of up to ∼47% of their starting force compared to ∼68% in age matched littermate controls [MD −22.01, 95% CI (−34.36, −9.66), *P* < 0.0001], whereas the 22-month-old mdx EDL muscles recovered the least with ∼25% of their starting compared to ∼65% in age matched littermate controls [MD −40.29, 95% CI (−52.88, −27.69), *P* < 0.0001]. The recovery in mdx EDL decreases as the dystrophic animal ages, resulting in a decline in end recovered force as shown in Figure 7C. Dystrophic EDL muscles at 9 months recovered ∼41% [MD −38.63, 95% CI (−51.06, −26.2), *P* < 0.0001], 15 months recovered ∼29% [MD −44.45, 95% CI (−59.08, −29.82), *P* < 0.0001] and 18 months recovered ∼33% [MD −39.08, 95% CI (−51.43, −26.73), *P* < 0.0001] of starting force. Given that both 4 and 22-month-old mdx mice contain 100% regenerated dystrophin-negative muscle fibers (Duddy et al., 2015), we attribute the large non-recoverable force loss in old mdx EDL to the increase in fiber branching (Figure 1).

In regards to twitch force recovery (Figure 7D), at 4 months of age, there were no significant differences between dystrophic and control muscles. This is a key finding, along with the fact that, at 4 months, there is no difference in either absolute or specific twitch force (Figures 3C,D), and the correlation with the low number and reduced complexity of branching at this age (Figure 1). However, the ability for EDL muscles from dystrophic animals to recover twitch force after our EC protocol decreased over the 9 to 22-month-old age groups (Figure 7D). EDL twitch force recovery for adult and aged groups are as follows: 9 months ∼30% for *mdx* compared to ∼54% for controls [MD −23.86, 95% CI (−36.7, −13.03), *P* < 0.0001], 15 months ∼20% for *mdx* compared to ∼45% for controls [MD −25.34, 95% CI (−37.49, −13.18), *P* < 0.0001], 18 months ∼22% for *mdx* compared to ∼44% for controls [MD −22.66% CI (−33.24, −12.07), *P* < 0.0001], and 22 months ∼17% for *mdx* compared to ∼51% for controls [MD −34.35, 95% CI (−23.02, −45.69), *P* < 0.0001]. Once again, it is striking that although 4 to 22-month-old dystrophic EDL muscles contain regenerated dystrophin negative fibers, the force loss and EC deficit is strongly correlated with the degree and complexity of branched fibers (Figure 1).

### Stiffness

**FIGURE 8:**
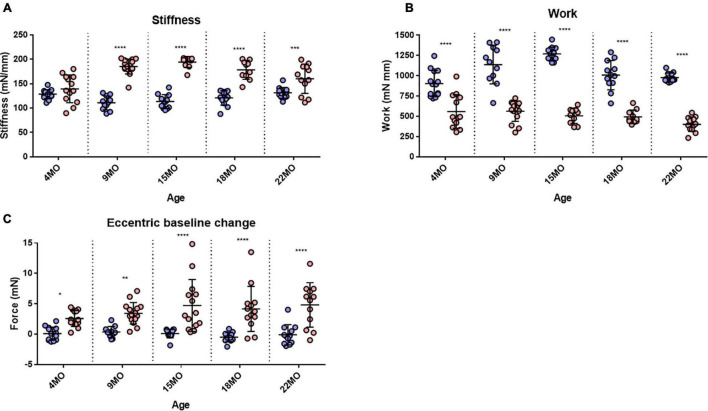
Stiffness, Work done and baseline force. **(A)** Interleaved scatterplots showing the EDL muscle stiffness calculated during the first 20% EC across 4-month (*n* = 14 control; *n* = 13 *mdx*), 9-month (*n* = 11 control; *n* = 15 *mdx*), 15-month (*n* = 12 control; *n* = 10 *mdx*), 18-month (*n* = 12 control; *n* = 10 *mdx*), and 22-month (*n* = 12 control; *n* = 12 *mdx*) age groups. **(B)** Interleaved scatterplots showing the work done by EDL muscles calculated during the first 20% EC across 4-month (*n* = 14 control; *n* = 13 *mdx*), 9-month (*n* = 11 control; *n* = 15 *mdx*), 15-month (*n* = 12 control; *n* = 10 *mdx*), 18-month (*n* = 12 control; *n* = 10 *mdx*), and 22-month (*n* = 12 control; *n* = 12 *mdx*) age groups. **(C)** Interleaved scatterplots showing the baseline force change post EC for *mdx* and littermate control EDL muscles across 4-month (*n* = 14 control; *n* = 13 *mdx*), 9-month (*n* = 11 control; *n* = 14 *mdx*), 15-month (*n* = 9 control; *n* = 14 *mdx*), 18-month (*n* = 12 control; *n* = 10 *mdx*), and 22-month (*n* = 12 control; *n* = 12 *mdx*) age groups. In each interleaved scatterplot group the horizontal line indicates the mean value ± SD. Statistical differences between age groups are displayed above the graph, statistical differences displayed within graphs are differences between genotypes assessed by two-way ANOVA, *post hoc* analysis using Sidak’s multiple comparisons test. ****P < 0.0001, ***0.0001 < *P* < 0.001, **0.001 < *P* < 0.01, and *0.01 < *P* < 0.05.

Stiffness is an inverse indication of muscle compliance. A stiffer muscle would exhibit a greater change in force for a given change in length. Our earlier skinned fiber studies of single non-branched muscle fibers from *mdx* mice 4–6 months of age reported no major differences in the function of contractile proteins (Williams et al., 1993). Here, the intact EDL muscle stiffness was calculated during the first EC and reported in Figure 8A. In the 4-month-old group, there was no significant difference in stiffness between *mdx* EDL and age matched littermate control EDL muscles. In contrast, age groups of 9–22 months show a significant increase in stiffness compared to age matched littermate control animals. The increase in stiffness is correlated with the increase in branching (Figure 1) and is likely the consequence of branching effectively increasing the number of small muscle fibers arranged in series within the aging dystrophic muscle. Differences in muscle stiffness between *mdx* and controls for each of these age groups are as follows: 9 months [MD 74.38, 95% CI (55.55, 93.21), *P* < 0.0001], 15 months [MD 80.97, 95% CI (60.66, 101.3), *P* < 0.0001], 18 months [MD 57.48, 95% CI (37.17, 77.78), *P* < 0.0001], and 22 months [MD 29.11, 95% CI (9.75, 48.47), *P* = 0.0007].

### Work Done

Several studies have reported that the work done to stretch the muscle during the lengthening phase of an EC was the best predictor of the magnitude of the force deficit produced (Brooks et al., 1995; Hunter and Faulkner, 1997; Stevens and Faulkner, 2000; Lindsay et al., 2020). We calculated the work done as the area under the force curve during the active lengthening phase of the EC (Figure 8B) and illustrates that the work done during the EC was significantly greater in age matched littermate controls compared with the dystrophic EDL muscle. Differences in work done between *mdx* and controls for each age group are as follows: 4 months [MD −334, 95% CI (−490.2, −197.8), *P* < 0.0001], 9 months [MD −574.9, 95% CI (−725.6, −424.2), *P* < 0.0001], 15 months [MD −763.5, 95% CI (−926, −600.9), *P* < 0.0001], 18 months [MD −514.1, 95% CI (−676.6, −351.6), *P* < 0.0001], and 22 months [MD −576.1, 95% CI (−731, −421.1), *P* < 0.0001]. It is interesting to note that less work is done on the *mdx* EDL compared with the work done on age matched littermate control EDL.

### Increase in Resting Force After the First Eccentric Contraction

The change in resting baseline absolute force after the first EC is shown in Figure 8C. In littermate control EDL muscles, baseline force remained at zero (the set point prior to the EC protocol) throughout different age groups following the first EC. EDL muscles from *mdx* mice produced a significantly higher resting force after an EC across all age groups, which increased in magnitude and spread with age. Differences in baseline change between *mdx* and controls for each age group are as follows: 4 months [MD 2.482, 95% CI (0.09, 4.88), *P* = 0.039], 9 months [MD 3.06, 95% CI (0.60, 5.53), *P* = 0.0077], 15 months [MD 4.61, 95% CI (2.23, 6.96), *P* < 0.0001], 18 months [MD 4.64, 95% CI (2.15, 7.13), *P* < 0.0001], and 22 months [MD 4.93, 95% CI (2.39, 7.47), *P* < 0.0001].

### Twitch Kinetics

As pointed out by Peczkowski et al. (2020), while maximal isometric tetanic contractions are most commonly used to assess and report muscle function *in vitro*, *in vivo* muscles likely contract at sub-maximal levels. To address this, we looked at twitch kinetics as an outcome parameter. Twitch kinetics were measured at (1) pre-eccentric, (2) post-eccentric, and (3) after recovery from the EC protocol with statistical analysis presented in Table 1. Twitch half relaxation times did not significantly change in littermate controls throughout all three measures across all age groups. In (1) pre-eccentric, this was the same for the dystrophic group with the exception of 4-month-old *mdx* EDL, which took significantly longer to relax. Following ECs, (2) post-eccentric twitch relaxation time for *mdx* EDL muscles increased in all age groups and were significantly higher than control values. When measured (3) after recovery from the EC protocol, dystrophic EDL muscles had reduced half relaxation time closer to starting values but still took significantly longer to relax relative to age matched littermate controls (see Table 1 for genotype statistics and distribution for kinetics).

Except for 4-month-old dystrophic EDLs, (1) pre-eccentric twitch time to peak measures showed that dystrophic EDL muscles had a significantly faster twitch time to peak compared to age matched controls in all age groups. In the 4-month-old group, both *mdx* and control muscle reached peak contraction at a similar time, likely due to the different levels of absolute force, see Figure 3C (if the muscle produces a greater twitch force it will take a longer time to achieve this when other parameters are the same). (2) Post-eccentric 4-month-old *mdx* EDL muscles TTP was significantly slower time than control counterparts. The time to peak for all older age groups remained similar with no differences between age or genotype. (3) After recovery from the EC protocol, dystrophic EDL muscles showed a significantly slower TTP than control animals at 4-, 9-, and 22-month age groups and remained similar for remaining cohorts (Table 1).

### Half Relaxation Time/Time to Peak Ratio for Twitch Kinetics

Peczkowski et al. (2020) developed the HRT/TTP ratio as a way of assessing twitch kinetics between genotypes and ages. Using this measure, littermate control EDL muscles, (1) pre-eccentric, (2) post-eccentric, and (3) after recovery (with the control EDL muscles contracting at a slower rate than they relax, HRT/TTP ratio less than 1), remain unchanged due to EC induced injury/recovery and stayed consistent across all age groups (Table 1). (1) Pre-eccentric HRT/TTP ratio was similar for dystrophic muscles compared to littermate controls in most age groups. (2) Post-eccentric HRT/TTP ratio increased for all *mdx* EDL muscles across all groups and remained elevated when measured (3) after recovery from the EC protocol.

The similar HRT/TTP ratio, (1) pre-eccentric in *mdx* and age-matched littermate controls suggest the fiber type profiles of the EDL remains unchanged in the *mdx*, while the higher HRT/TTP ratio reported for dystrophic muscle fibers post EC shows dystrophic muscles contracting at a similar rate to relaxing. This might be due to the presence of stressed branched fibers with reduced excitability and slower rates of contraction and relaxation throughout the branched syncytium.

### Light Microscope Morphology of Enzymatically Isolated Single Fibers

**FIGURE 9:**
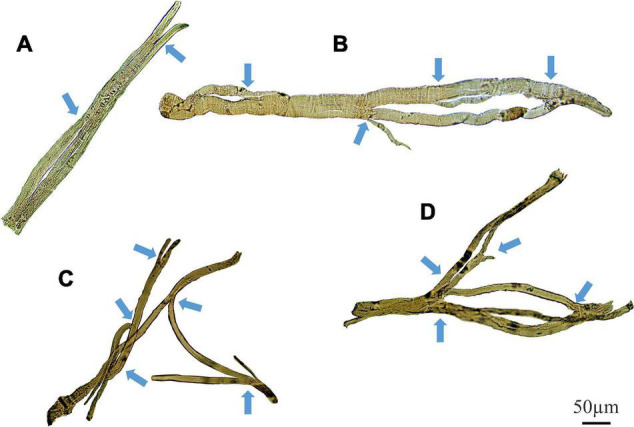
Examples of medium power light microscope images (X100) of enzymatically digested EDL muscle fibers taken from a single 22-month-old dystrophic mouse. Note the fibers have been stitched together at the same magnification from photomicrographs taken from overlapping fields of view to capture a large portion of the fiber, scale bar provided at 50 μm. Backgrounds debris from the digest process have been cleared to focus on the muscle fiber and arrows mark areas where fiber branching has occurred (To see examples where debris have not been removed see Kiriaev et al. (2018). **(A)** A fiber containing a single branch with slits forming within the trunk. **(B)** A fiber showing rejoining of these slits within the trunk and multiple offshoots off the edge of the muscle. **(C,D)** Examples of complex branched muscle fibers.

Representative stitched images of intact muscle fibers, taken at magnification (X100) on a light microscope, demonstrate various degrees of complex fiber branching in the senescent (22 month) *mdx* EDL (Figure 9). Figure 9A shows an example of a simple branched fiber containing one branched end and multiple splits within itself that develop along the length of the fiber. These splits along the fiber can become quite large and more noticeable such as in Figure 9B where we can see several branched offshoots from the main fiber trunk. Figures 9C,D show examples of complex branching (4+ branches) with multiple offshoots along the length of the dystrophic fiber. Major branch points have been marked with arrows for each fiber.

**FIGURE 10:**
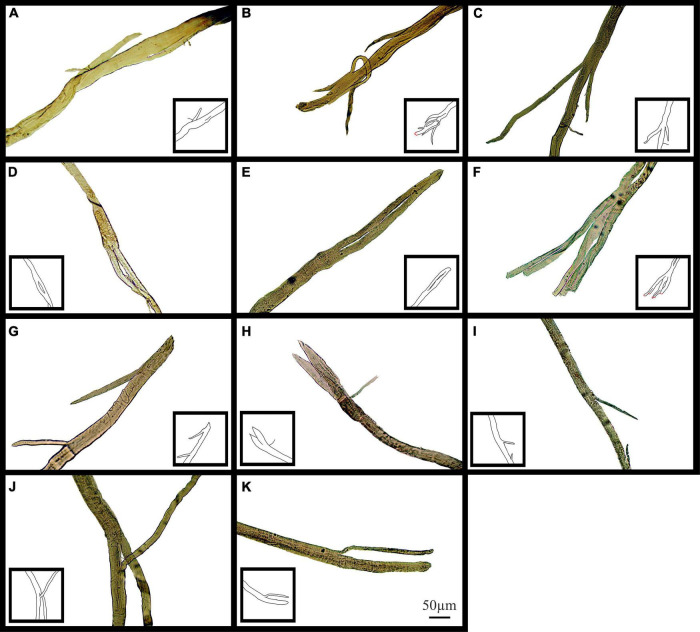
Examples of medium power light microscope images (X100) of muscle fiber branch point sections that have not undergone EC from adult and senescent *mdx* mice. Cartoon inserts have been added to help visualize the various examples of branching in each image. **(A–F)** Are portions of fibers from a 22-month *mdx* mouse EDL. **(G–K)** Are portions of fibers from an 18-month *mdx* mouse EDL. Scale bar provided at 50 μm in **(K)** applies to all of figure.

Figure 10 shows examples of (single field X100) light microscope images taken of branch points in aged *mdx* EDL muscle fibers before EC. The branch pattern in these *mdx* fibers range from offshoots from the main trunk of the fiber shown in Figures 10A,C,G,I,J to splits that rejoin mid fiber in panels Figures 10D,E along with branching toward the end of fibers in Figures 10B,F,H,K.

**FIGURE 11:**
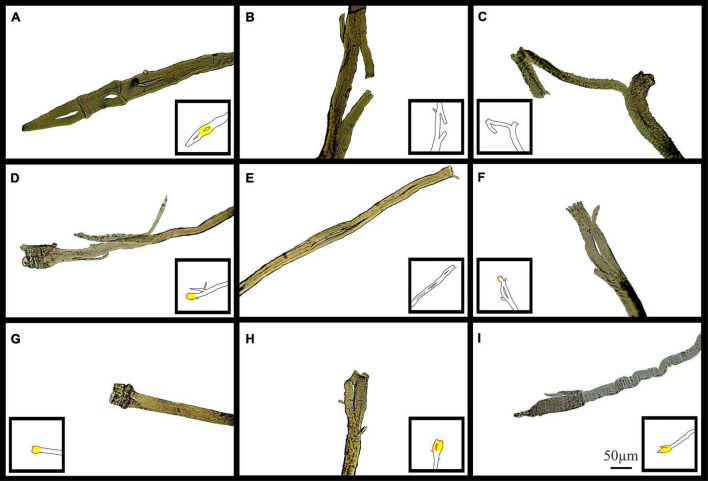
Examples of medium power light microscope images (X100) of broken muscle fiber sections that have undergone ECs from adult and senescent *mdx* mice. Broken areas have been outlined in red whilst swollen and necrotic areas highlighted yellow in cartoon inserts for each image. **(A–D)** Are portions of fibers from a 22-month *mdx* mouse EDL. **(E–I)** Are portions of fibers from a, 18-month *mdx* mouse EDL. Scale bar provided at 50 μm in **(I)** applies to all of figure.

Figure 11 shows typical pictures of *mdx* fiber branching in relation to mechanical findings post EC. These photomicrographs illustrate, in a qualitative manner, breaks in branched fibers, which we propose occurred because of the EC. In Figure 11, line drawing inserts have been added to each image to show the areas we have hypothesized as having broken during EC (Red) or have become hypercontracted due to EC rupture (Yellow).

## Discussion

**FIGURE 12:**
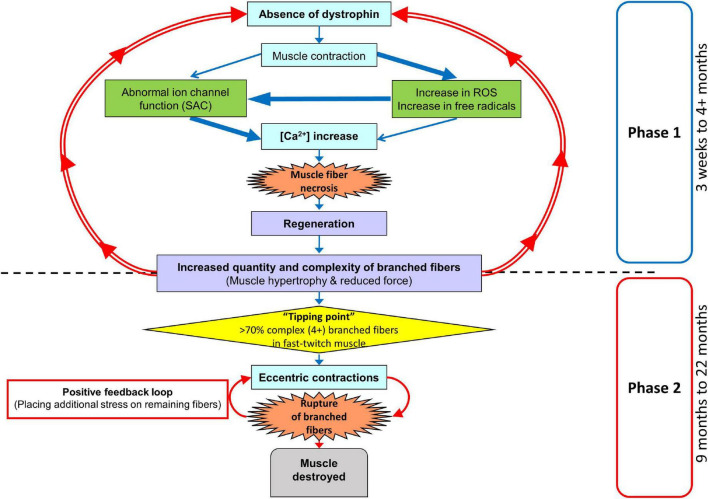
Two-phase model of disease pathogenesis in *mdx* mice. Phase-one involve the absence of dystrophin triggering skeletal muscle fiber necrosis, driven by a pathological increase in [Ca^2+^]_in_. The process is likely caused by a combination of increased free radical damage and abnormal ion channel functioning during muscle contraction. Phase-one involves repeated cycles of muscle fiber regeneration and necrosis leading to the development of abnormally branched regenerated dystrophin-deficient muscle fibers. These branches increase in quantity and complexity with age as the number of repeated regenerative cycles in phase-one increase. Once the number and complexity of the branched fibers passes a level we have termed “tipping point,” phase-two is initiated. Now, the fiber damage is a consequence of the weak branched fibers rupturing, particularly during eccentric contractions. This will tend to have positive feedback as the broken branches will no longer support the contracting muscle placing additional stress on the remaining branches. In the final period of phase-two the majority of muscle tissue will be terminally damaged beyond repair. It is important to note, that depending on the forces experienced by the muscle, phase-one and phase-two can occur at the same time.

The fast-twitch EDL skeletal muscle from the *mdx* mouse is the muscle most used to study the pathophysiology caused by an absence of dystrophin from the inner surface of the sarcolemma. The mouse EDL is a mix of fast fiber types; ∼79% type 2B (fast glycolytic), ∼16% type 2X and ∼4% type 2A (fast oxidative glycolytic; Hettige et al., 2020). We have previously proposed a two-stage model to describe the skeletal muscle pathology in the dystrophinopathies (Chan et al., 2007; Head, 2010, 2012; Chan and Head, 2011; Kiriaev et al., 2018). Here, we extend our model to take account of our new data (see Figure 12). We now term the stages as phases. Phase-one involves the absence of dystrophin triggering skeletal muscle fiber necrosis driven by a pathological increase in [Ca^2+^]_in_, likely caused by a combination of increased free radical damage and abnormal ion channel functioning during muscle contraction. Phase-one is cyclic, with repeated cycles of muscle fiber regeneration and necrosis. In phase-one, the regenerated dystrophin-deficient muscle fibers are abnormally branched, and this branching pathology increases in complexity with age as the number of regenerative cycles in phase-one increases. This abnormal fiber branching is responsible for the hypertrophy and reduced maximal force output characteristic of older *mdx* EDL. Once the number and complexity of the branched fibers passes a level we have termed “tipping point,” phase-two is initiated. Now, the fiber damage is a consequence of the weak branched fibers rupturing, particularly during ECs, and this will tend to have positive feedback as the broken branches will no longer support the contracting muscle placing additional stress on the remaining branches. In the final period of phase-two, the majority of the muscle tissue will be damaged. It is important to note that depending on the forces experienced by the muscle, phase-one and phase-two can occur at the same time. Here, we have provided further evidence to test this two-phase model by looking at the correlation between fiber branching and the contractile pathology in dystrophin-deficient EDL muscles from *mdx* mice between 4 and 22 months of age compared with age matched littermate controls. By 4 months of age, 100% of *mdx* skeletal muscle fibers have undergone at least one round of necrosis/regeneration (Duddy et al., 2015), so all the contractile findings reported here are from regenerated dystrophin deficient fast-twitch fibers. With reference to DMD, our 4 and 9-month-old mice can be considered representative of the adolescent population while 15, 18, and 22-month-old represent adults.

### Fiber Branching

Dystrophic EDL muscles between 4 and 22 months are comprised of 100% regenerated dystrophin-negative muscle fibers (Carnwath and Shotton, 1987; Coulton et al., 1988; Pastoret and Sebille, 1995; Kornegay et al., 2012; Faber et al., 2014; Pichavant and Pavlath, 2014; Duddy et al., 2015), the major morphological change that occurs during this period is the formation of branched fibers which increase in number and complexity as the animal ages (Figures 1, 9; Head et al., 1992; Chan et al., 2007; Lovering et al., 2009; Pavlath, 2010; Chan and Head, 2011; Faber et al., 2014; Pichavant and Pavlath, 2014; Duddy et al., 2015; Kiriaev et al., 2018). All the contractile deficits and increased dystrophic muscle weight we report in this study are correlated with the increase in quantity and complexity of these branches. The reported increase in active stiffness (Figure 8A) with age in the *mdx* EDL is a direct consequence of the increase in number and complexity of branched fibers. We calculate stiffness as the change in force divided by the change in length. This means that shorter fibers will appear stiffer because they undergo a greater strain for a given muscle deformation (Patel and Lieber, 1997). We have shown, in our earlier paper, that in the intact old *mdx* EDL muscle, the short branches present on each fiber lie longitudinally within the muscle (Kiriaev et al., 2018). This means that branching effectively increases the number of shorter fibers present in the muscle, which directly increases the active stiffness of the muscle due to the cross-bridge biomechanical properties. In humans, it has been proposed that skeletal muscle fiber branching is a key pathological sign of the progressive muscular dystrophies (Pearson, 1965; Pichavant and Pavlath, 2014). Branched skeletal fibers are found in the muscles of boys with DMD (Pearce and Walton, 1962; Bell and Conen, 1968; Schwartz et al., 1976; Swash and Schwartz, 1977; Schmalbruch, 1984; Ben Hamida et al., 1992; Cooper and Head, 2014). In DMD boys, extensive fiber branching has been correlated with a reduction in mobility (Bell and Conen, 1968). Since our first report (Head et al., 1992) of fiber branching in the *mdx* hindlimb muscles, this finding has been confirmed by our laboratory and others (Chan et al., 2007; Lovering et al., 2009; Friedrich et al., 2010; Pavlath, 2010; Chan and Head, 2011; Buttgereit et al., 2013; Faber et al., 2014; Pichavant and Pavlath, 2014; Duddy et al., 2015; Kiriaev et al., 2018).

### Hypertrophy and Loss of Force in Fast-Twitch *mdx* Muscles

Skeletal muscle hypertrophy is a characteristic of the dystrophinopathies (Bell and Conen, 1968; Jones et al., 1983; De Visser and Verbeeten, 1985; Cros et al., 1989; Reimers et al., 1996; Tyler, 2003; Marden et al., 2005; Head, 2012; Kornegay et al., 2012). In the mouse *mdx* EDL, we and others have reported a 20–30% hypertrophy in EDL (Coulton et al., 1988; Pastoret and Sebille, 1993; Faulkner et al., 1997; Lynch et al., 2001a; Chan et al., 2007; Hakim et al., 2011; Hakim and Duan, 2012; Faber et al., 2014; Duddy et al., 2015; Kiriaev et al., 2018; Massopust et al., 2020) while reports of 20–60% hypertrophy have been published regarding the predominantly fast-twitch *mdx* TA (Quinlan et al., 1992; Pastoret and Sebille, 1995; Dellorusso et al., 2001; Froehner et al., 2014; Massopust et al., 2020; Ben Larbi et al., 2021). In the *mdx* mouse, this hypertrophy initially maintains the absolute force output of the *mdx* EDL at 4 months, while by 9–22 months, despite the larger muscles, the absolute force drops relative to age matched littermate control muscles (Figures 3A,C). In terms of maximum specific force, the *mdx* EDL produces less at all ages (Figure 3B) compared to littermate controls and this difference increases with age, being most marked in the 22-month *mdx* EDL muscles. The picture is similar for the specific twitch force apart from the 4-month group where there is no difference (Figure 3D). Given the strong correlation between the increase in branched fibers, hypertrophy, and lower force output at 9–22 months of age in the *mdx* mouse, we propose that pathological fiber branching is responsible for both the reduction in force output and hypertrophy. The correlation between branching and force loss is also maintained when we look at the fitted force frequency curves (Figure 4) where the drop of force in *mdx* fibers is correlated with the increase in branched fibers (Figures 1, 9, 10). Statistically, there is no change in the half frequency or the Hill coefficient of the curves (Table 1), suggesting that, functionally, the *mdx* EDL muscles have remained fast-twitch during regeneration (4–22 months). This confirms studies that have reported no significant changes in fiber types with age in *mdx* muscle (Carnwath and Shotton, 1987; Anderson et al., 1988; Williams et al., 1993; Hayes and Williams, 1998; Chan et al., 2007; Kiriaev et al., 2018). In the *mdx* limb muscles, fiber branching itself has been shown to be the major component of muscle hypertrophy which occurs as the *mdx* mouse ages (Faber et al., 2014; Pichavant and Pavlath, 2014; Duddy et al., 2015). Faber et al. (2014) demonstrates that, in dystrophic EDL muscles, a 28% increase in the number of fibers counted in transverse sections of muscles correlated with a 31% increase in myofiber branching. Furthermore, the study highlights the largest increase in myofiber number and branching both occurred at 12 weeks onward, confirming through histology a phase of severe degeneration occurring just prior. Recently, a lifetime analysis of *mdx* skeletal muscle performed by Massopust et al. (2020) confirmed the findings of Faber et al. (2014) which discussed that myofiber diameter and volume develop normally until 12 weeks of age where dystrophic muscles become significantly larger than age matched controls. Importantly, the study identified that muscle fiber branching contributes to volume, diameter, and CSA variability and that branches themselves do not feature synapses, thus receiving innervation from the original fiber (Faber et al., 2014; Duddy et al., 2015; Massopust et al., 2020).

### Why Does Fiber Branching Reduce Force Output in *mdx* Muscles?

Friedrich’s laboratory (Friedrich et al., 2010) have produced a convincing structural hypothesis where they propose the repeated bouts of regeneration and degeneration in *mdx* muscles produce branched fibers which have been structurally remodeled in such a way that contractile proteins have become misaligned and deviate from the long axis of the muscle, leading to asynchronized contraction and loss of force production. Furthermore, within a regenerated dystrophic fiber, the group used second harmonic generation imaging to identify an increased variation in the angle of myofibril orientations (Buttgereit et al., 2013) which, they calculate, could contribute to ∼50% of the progressive force loss with age in *mdx*. Additionally, several studies have confirmed that each branched fiber syncytium, no matter how many branches they contain (the most complex in excess of 10), is controlled by a single neuromuscular junction (a single motor nerve; Faber et al., 2014; Pratt et al., 2015; Massopust et al., 2020). We have modeled (Head, 2010) the transmission of a muscle action potential through a syncytium, showing it will be slowed in small diameter branches and can fail to transmit when propagation is from a small diameter branch toward a large diameter branch. This means that the force output per cross-section of branched fiber syncytium will be reduced by the number of non-contracting small branches. Thus, we propose that the correlation of increased fiber branching with hypertrophy and decreased force output in dystrophin deficient muscles is not the result of the absence of dystrophin. Rather, it is due to the absence of dystrophin triggering rounds of necrosis/regeneration which then produces branched fibers that lead to muscle hypertrophy and reduce force output (Figure 12). Further evidence in support of this comes from our skinned fiber studies (Head et al., 1990; Head, 2010) where we chemically remove the cell membrane (sarcolemma), thus removing the dystrophin protein link in control muscles and examine the force output from non-branched segments of contractile proteins from *mdx* EDL compared to controls. We showed that there is no difference in the force output produced by these contractile proteins.

### Repeated Maximal Isometric Contractions in Extensor Digitorum Longus From 18 and 22-Month-Old *mdx* Mice Produce a Non-Recoverable Force Deficit Which Is Correlated With the Number and Complexity of Branched Fibers

When we gave the EDL 10 maximal isometric contractions, separated by rest periods of 60 s to reduce the impact of fatigue. It was only the 18 and 22-month-old *mdx* EDL which showed a significant loss of force that did not recover. Our argument can be summarized using Figures 5B, 12 where during isometric contractions 4-month-old dystrophin deficient EDL have a similar force deficit and recovery to EDL from age matched littermate controls. In contrast, 22-month-old dystrophin deficient muscles with extensive complex branching (Figures 1, 9) only generate around ∼48% of their starting force by the 10th isometric contraction which recovered to ∼60% of maximal. These maximal isometric forces are unlikely to be experienced *in vivo*. Nonetheless, it supports our contention that extensively branched *mdx* fast-twitch EDL muscle fibers cannot sustain maximal isometric forces due to the branch points mechanically weakening the fiber (Figures 10–12). Claflin and Brooks (2008) showed that 5 to 13-month *mdx* lumbrical muscles experience an irreversible loss of force when subjected to 10 isometric contractions. However, they did not look at fiber branching. When modeling stretching of a muscle fiber Iyer et al. (2017) showed non-uniform strain distributions at branch points in single fibers, whereas uniform strain distribution was observed in fibers with normal morphology and concluded this increased susceptibility to stretch-induced damage occurring in branched myofibers at the branch point. Further evidence that extensive branching structurally weakens the dystrophic fiber, making it prone to rupture when producing high forces, comes from using the skinned muscle fiber technique to study single branched fibers. When a branched dystrophic skinned fiber was attached to a force transducer, the fiber commonly breaks at a branch point when exposed to a series of solutions with increasing [Ca^2+^]. Importantly, when the remaining non-branched segment of the same fiber was reattached, it could sustain maximal [Ca^2+^] activated force (Head et al., 1990; Head, 2010). Additional evidence from past studies performed on merosin deficient dystrophic mice with extensive branching have shown that the branched fibers are damaged by isometric contractions (Head et al., 1990). It is possible that during the repeated isometric contractions, these *in vivo* muscles, devoid of a blood supply, may develop an anoxic core. Modeling by Barclay’s group show muscles of the same diameter used at room temperature, with saturated O_2_, and 60 s duty cycle as used here will not develop a significant anoxic core (Barclay, 2005; Barclay et al., 2010).

### Reversible and Irreversible Recovery of Force Deficit Post Eccentric Contractions

In Figures 6A–C, we show that 9 to 22-month-old *mdx* with extensive complexed branching (Figures 1, 9) have a catastrophic force deficit on the first of six ECs. The 4-month *mdx* have a graded force loss over the six ECs which, though more marked, is similar in profile to controls (Figures 6A,B). We attribute the catastrophic force drop on the first EC in older 9 to 22-month-old *mdx* (Figure 6C) to the structural weakness cause by the presence of large numbers of complexed branched fibers (Figures 1, 9) present in the 9 to 22-month-old dystrophic EDL. Previously, we and others, have proposed that extensively complex branching structurally weakens fast-twitch fibers (Pearce and Walton, 1962; Bell and Conen, 1968; Schwartz et al., 1976; Swash and Schwartz, 1977; Schmalbruch, 1984; Head et al., 1990, 1992, 2004; Ben Hamida et al., 1992; Chan et al., 2007; Lovering et al., 2009; Friedrich et al., 2010; Pavlath, 2010; Cooper and Head, 2014; Pichavant and Pavlath, 2014; Kiriaev et al., 2018). In Figure 6B, both 4 and 22-month *mdx* are dystrophin deficient but as the animals age, the absence of dystrophin triggers cycles of necrosis/regeneration and an age-related increase in complex branched fibers which, we propose, rupture on the first EC (Figure 6B lower trace and Figures 1, 9, 11, 12). Olthoff et al. (2018) showed in 3-month-old *mdx* EDL muscles that there was a graded 90% force deficit which occurred over 10 EC given at 3 min intervals. The authors attributed the bulk of this force deficit to mechanisms linked to pathological ROS production in the dystrophic 3-month EDL muscles because there was a 65% recovery of force within 120 min. When EC cycles were delivered at 30-min intervals, there was also a reversible loss of force. Their study concluded that the EC force deficit in dystrophic EDL is not due to sarcolemmal rupturing, as would be the case if dystrophin was acting as a shock absorber. Instead, Olthoff et al. (2018) proposed an alternative hypothesis, that ECs in their EDL muscles from 3-monthold mice drives a transient, redox-based inhibition of contractility. Lindsay et al. (2018) demonstrated a similar result, again in 3-month *mdx* EDL muscles, where following 75% eccentric force deficit, dystrophic muscles recovered up to 64% at 60 min post contraction. In support of the redox hypothesis, when Lindsay et al. (2018) added the macrophage synthesized antioxidant 7,8-Dihydroneopterin to the 3-month-old *mdx* muscles, it provided protection against ECs and improved force recovery to 81%. The authors conclude that the restoration of isometric tetanic force with antioxidant treatment in *mdx* muscle suggest reversible oxidation of proteins regulating muscle contraction. Both these cases and other studies report force recovery post-EC in fast-twitch muscle from young *mdx* mice (3–12 weeks of age; Han et al., 2011; Call et al., 2013; Roy et al., 2016). According to our two-phase model of damage in the dystrophinopathies, dystrophic fast-twitch fiber branching had not reached “tipping point” in phase-two, and the majority of the fast-twitch fibers are cycling in phase-one (Figure 12). When the “tipping point” is passed, there is an irrevocable sarcolemma rupture at branch points with positive feedback, to the effect that branches rupture so it increases the strain on the remaining branches. In our 2018 paper, we showed it was only in old *mdx* EDL with extensively branched fibers that there was a loss of ∼65% of the force on the first EC which did not recover (Kiriaev et al., 2018). Here, we have confirmed this finding and extended it by looking at recovery post-EC at 120 min and by showing that in 4-month-old *mdx* EDL muscles, (with considerably less branching) there is a graded force loss over the six EC which recovers by ∼47% after 120 min compared to only a ∼25% recovery in the old dystrophic EDLs with extensively complexed branching. It should be noted that EC stretches are specified relative to *L*_*o*_ while uncertain of fiber length, the fiber strain during these stretches may be greater than the 20% indicated where our EC protocol is severe enough to produce a non-recoverable force deficit of 20–35% in age matched littermate controls. Figure 8C shows an age-related increase in baseline force after EC, likely due to the increasing number of branched fibers becoming damaged, leaving some fibers with a high resting [Ca^2+^]_in_ which produces an increase in resting tension. Our current findings, along with the review by Allen et al. (2016) and recent work mentioned (Lindsay et al., 2018, 2020; Olthoff et al., 2018), add to the growing body of evidence in support of our two-phase hypothesis to explain the pathology of the dystrophinopathies (Chan et al., 2007; Head, 2010, 2012; Chan and Head, 2011; Kiriaev et al., 2018).

## Summary

In the present study, we have demonstrated that *mdx* EDL muscles from adult to senescent age groups have the largest component of eccentric damage which occurs as an abrupt loss of force on the first EC (Figures 6A–C). As previously reported, we propose that this is the result of mechanical rupture of branched dystrophic fibers (Dellorusso et al., 2001; Kiriaev et al., 2018). In contrast, younger adolescent *mdx* muscle have a graded drop in force during each phase of the EC protocol (Roy et al., 2016; Capogrosso et al., 2017), resembling those seen in littermate control muscles (Figures 6A,B). When dystrophic muscles are left to recover 120 min post EC, we also report rapidly reversible recovery of EC force deficit in young adolescent animals but to a lesser degree [∼47% compared to ∼65% in Olthoff et al. (2018)] than those published previously (Figures 7C,D). Given that Lindsay et al. (2020) reported that the EC strain is the major predictor of the force deficit, differences in the present study are likely attributable to the increased magnitude in EC strain we used (Figure 8B). In aged and senescent dystrophic animals, recovery is only ∼20–30% of starting force (Figure 7), indicating a smaller reversible force loss component and a larger irreversible component due to acute membrane rupture. We attribute this irreversible component to the presence of complex branches in aged and senescent *mdx* mice which are prone to rupture following EC induced injury becoming functionally obsolete. Dependent on the EC strain, degree of branching and complexity of branching, the capacity for recovery varies in *mdx* muscle and can explain the variability reported in literature (Head et al., 1992; Moens et al., 1993; Brooks, 1998; Han et al., 2011; Call et al., 2013; Roy et al., 2016; Capogrosso et al., 2017; Lindsay et al., 2018; Olthoff et al., 2018). Our current findings highlight the importance of studying the muscle pathophysiology in the *mdx* dystrophin-deficient mouse at all age points throughout the dystrophic animals’ life span. Many studies report skeletal muscle pathologies in *mdx* mice between 6 and 12 weeks of age yet fail to address why these dystrophin-deficient animals can live largely asymptomatically past 108 weeks of age. It is only in the old *mdx* mouse that we start to see them die earlier than age matched controls (Chamberlain et al., 2007; Li et al., 2009) and we correlate this with the degree and complexity of muscle fiber branching where a significant proportion of branches can no longer withstand the normal stresses and strains of muscle contraction.

## Data Availability Statement

The raw data supporting the conclusions of this article will be made available by the authors, upon reasonable request.

## Ethics Statement

The animal study was reviewed and approved by Western Sydney University Animal Care and Ethics Committee.

## Author Contributions

LK, SH, PH, JM, and KN conceived and designed the experiment. LK and SK collected the data. LK and SH performed the analysis and wrote the manuscript. LK, SH, SK, PH, JM, and KN revised and approved the manuscript. All authors contributed to the article and approved the submitted version.

## Conflict of Interest

The authors declare that the research was conducted in the absence of any commercial or financial relationships that could be construed as a potential conflict of interest.

## Publisher’s Note

All claims expressed in this article are solely those of the authors and do not necessarily represent those of their affiliated organizations, or those of the publisher, the editors and the reviewers. Any product that may be evaluated in this article, or claim that may be made by its manufacturer, is not guaranteed or endorsed by the publisher.
